# Dispersion of *Leishmania (Leishmania) infantum* in central-southern Brazil: Evidence from an integrative approach

**DOI:** 10.1371/journal.pntd.0007639

**Published:** 2019-08-29

**Authors:** Aline Kuhn Sbruzzi Pasquali, Rafael Antunes Baggio, Walter Antonio Boeger, Nilsa González-Britez, Deborah Carbonera Guedes, Enmanuel Céspedes Chaves, Vanete Thomaz-Soccol

**Affiliations:** 1 Laboratório de Biologia Molecular, Departamento de Engenharia de Bioprocessos e Biotecnologia, Universidade Federal do Paraná, Brazil; 2 Programa de Pós-Graduação em Engenharia de Bioprocessos e Biotecnologia, Universidade Federal do Paraná, Brazil; 3 Laboratório de Ecologia Molecular e Parasitologia Evolutiva, Departamento de Zoologia, Universidade Federal do Paraná, Brazil; 4 Departamento de Medicina Tropical, Instituto de Investigaciones en Ciencias de la Salud, Universidad Nacional de Asunción (IICS-UNA), Paraguay; Institut Pasteur de Tunis, TUNISIA

## Abstract

*Leishmania (Leishmania) infantum* is the zoonotic agent of visceral leishmaniasis (VL), a disease with a global distribution. The transmission scenario of VL has been undergoing changes worldwide, with the biologic cycle invading urbanized areas and dispersing the parasites into other previously free areas. The epidemiological cycle in Brazil has dispersed from the Northeast to other regions of the country. In this study, an integrative approach, including genotyping Brazilian strains of *L*. *(L*.*) infantum* for 14 microsatellite markers and reviewing historical records of the disease, was used to assess dispersion routes throughout central-southern Brazil. Our results support three *L*. *(L*.*) infantum* dispersion routes: A) dispersion from Bolivia to the states of Mato Grosso, Mato Grosso do Sul and São Paulo via the Bolivia-Brazil gas pipeline from 1998 to 2005; B) VL dispersion from Paraguay to the Brazilian side of the triple border (Foz do Iguaçu and Santa Terezinha de Itaipu) during after 2012; and C) emergence of a new *L*. *(L*.*) infantum* cluster in western Santa Catarina State and its dispersion to southern Paraná State (municipality of Pato Branco), after 2013. Hypotheses regarding possible entries of *Leishmania (L*.*) infantum* into the area of the triple border are presented and discussed. Understanding how VL has dispersed is vital to the development of control measures for this disease and to avoid future dispersion events.

## Author summary

The dispersion of visceral leishmaniasis is an enigma. The State of Paraná, in southern Brazil, borders the states of São Paulo and Mato Grosso, which have experienced LV epidemics over the past 20 years. Therefore, we expected that the disease would enter this state through the contiguity of epidemics from other regions following by "ghost shadows". However, in 2012, the vectors of the parasite were reported in the western region (Foz do Iguaçu) of Paraná state, far from the epidemic regions. In the cross-sectional study, 23.8% of the dogs were infected, which is more than the eyes can see, showing an unexpected scenario where the disease was already widespread in the city. Now the question was: where does the life cycle element came from? In this study, we used genetic markers to understand the dispersion of *Leishmania infantum* throughout central-southern Brazil. Our results showed two possible agent inputs in the Paraná state, one coming from Paraguay and, another from Santa Catarina state. When we verify our results we perceived the monitoring importance of the distribution of these agents by diverse hypotheses, not only those that the scientific literature presents. Another relevant factor is always to be attentive to the environmental and socioeconomic events that can provide this dispersion.

## Introduction

Human Visceral Leishmaniasis (hVL) is a widely distributed neglected disease caused by the protozoans *Leishmania* (*Leishmania*) *infantum* in Asia, Africa, Europe and Americas, and *L*. (*L*.) *donovani* in Asia and Africa [1]. These parasites use the domestic dog as a reservoir, in which it causes canine Visceral Leishmaniasis (cVL), and Phlebotominae sand fly species of *Phlebotomus* and *Lutzomyia longipalpis* as vectors in the Old and New World, respectively [2], although other phlebotominae species have been hypothesized as secondary vectors in the latter region (see Thomaz-Soccol et al. [3] for further discussion).

VL has recently experienced changes in its transmission profile in both the Old and New World [1,4–6]. The disease has dispersed to places where it had not been previously described (e.g. United States, Uruguay, Madrid Spain), and has expanded its geographical distribution into previously free areas in endemic countries [7–11]. Thus, the number of cases of VL has increased in recent last years in both the Old (e.g. [6,12]) and New World [e.g. [13,14]. Currently, 1.69 billion people are estimated to be living in VL transmission areas worldwide, the disease presented 2.27 cases per 100,000 habitants in 2015, and 90% of global VL cases occurred in six countries, including Brazil [14–16].

Although known since 1913 [17] *L*. *(L*.*) infantum* is likely an invasive species in Brazil, arriving first in the Northeast Region carried by dogs transported with colonizers from Portugal and Spain [18–21]. Between 1920 and 1980, VL was restricted to rural areas in Northeast Brazil, where it has remained endemic [4, 22–24]. However, the disease subsequently began to invade urban and peri-urban areas in other regions of the country [25–29], with epidemics in the north region, especially in Teresina, state of Piaui, in 1981 and in São Luis, state of Maranhão, in 1982 [30,31]. In the subsequent decade, several epidemic outbreaks were reported, especially in the Southeast and Central-West regions, with high rates of cVL cases followed by clinical human cases in Belo Horizonte, state of Minas Gerais, Campo Grande, state of Mato Grosso do Sul, and Araçatuba, state of São Paulo. Now, *L*. *(L*.*) infantum* has spread throughout the states of Minas Gerais, Goiás, São Paulo, Mato Grosso, Mato Grosso do Sul, Rio de Janeiro and Espírito Santo [1,4, 26,32–37]

Dispersion of VL in the Southern Region of Brazil has been more recent. The first records of cVL and hVL in this region were in the state of Rio Grande do Sul in 2006 and 2008, respectively [38], followed by the state of Santa Catarina in 2011 [39,40]. In the state of Paraná the first detection of vectors and dogs diagnosed with cVL was in 2012, while the first human case was recorded in 2016 [41–45]. In the South Region of Brazil, VL occurs primarily in cities bordering Paraguay, Argentina and Uruguay [3, 9–11,46]. Currently, the disease is classified as ‘controlled’ in Brazil according with to its epidemiological scenario [47].

Several hypotheses have been proposed for the spread of *L*. *(L*.*) infantum* throughout central-southern Brazil. For instance, the construction of the east-west route of the Bolivia-Brazil gas pipeline is thought to have allowed the dispersion of *L*. *(L*.*) infantum* into the central-southern Brazil through the migration of workers and infected dogs and deforestation in the 1990s [1,48–53]. Moreover, the construction of railways and the immigration of infected dogs from other endemic areas seemed to have also facilitated the spread of the disease throughout central-southern Brazil [36, 50, 52, 54]. Deforestation and climate and environmental changes have also been proposed as assisting the expansion of VL in different parts of Brazil [22,49]. Most of these studies used different data to test these hypotheses, including molecular markers (i.e. microsatellite markers, see Ferreira et al. [51]) and historical spatial data (e.g. [49,50,55]). However, no study has tested these hypotheses using an integrative approach that combines both methods.

As part of the IDRC #107577–002 research project (idrc.ca/en/project/ addressing-emergence-and-spread-leishmaniasis-bordersargentina-brazil-and-paraguay), the objective of the present study was to evaluate the dispersion of *L*. *(L*.*) infantum* in central-southern Brazil by integrating molecular markers and historical records for hVL and cVL in the region. Assessing which cluster of *L*. *(L*.*) infantum* is present in each city allows reconstructing potential dispersion routes, while integrating spatial-temporal analysis of the first descriptions of VL cases helps to determine the direction of dispersion. Knowing dispersion routes is essential for developing strategies to control this emergent disease and restrain its future dispersion.

## Materials and methods

### Samples

One hundred and thirty-two isolates from dogs, humans and sand flies were genotyped for 14 loci of microsatellite markers to assess the dispersion of *L*. *(L*.*) infantum* in central-southern Brazil (Table 1). For this study, seventy samples were collected in four areas in the municipality of Foz do Iguaçu, Paraná (62 from dogs, four from sand flies and four from humans), and four samples from dogs collected in Santa Terezinha de Itaipu, Paraná, between 2013 and 2016 (see Thomaz Soccol et al. [45] for collection and parasite isolation details). Briefly, *Leishmania* strains from Foz do Iguaçu and Santa Terezinha de Itaipu were isolated from bone marrow, aspiration of lymph nodes and leukocyte layer of dogs, intestines of sand flies, and leukocyte layer of humans with clinical symptoms. These samples were inoculated in Neal, Novy and Nicole (NNN) culture medium with 0.9% saline solution for four weeks at 24ºC [56]. The promastigote cultures from the other samples were cultivated in Brain Heart Infusion (BHI) with 0.9% saline solution at 24ºC. After culture, parasites were centrifuged at 3,500 *g* at 4°C and washed three times (0.9% saline solution, 0.3% saline solution and again 0.9% saline solution). The DNA of cultured promastigotes and biological samples was extracted using the phenol/chloroform/isoamyl alcohol method [57].

**Table 1:** Number of strains (N), gene diversity (H), inbreeding coefficient (Fis) and allelic richness of 10 microsatellite markers of strains from 16 *L*. *(L*.*) infantum* populations in central-southern Brazil, one from Paraguay and one from the Old World.

Population (Code—City, State)	N	H	Fis	Allelic richness										
				Li 46–67	Li 71–7	Li 71–33	Li 23–41	Li 22–35	Lm2TG	Lm4TA	CS20	Li 71-5/2	List 7039	Mean
CE–Fortaleza, Ceará	1													
MG—Belo Horizonte, Minas Gerais	8	0.00	**	1.0	1.0	1.0	1.0	1.0	1.0	1.0	1.0	1.0	1.0	1.0
MS_1_—Três Lagoas, Mato Grosso do Sul	2													
MS_2_—Campo Grande, Mato Grosso do Sul	3													
MT–Rondonópolis, Mato Grosso	1													
OW—Old World	7	0.58	0.71*	2.0	2.4	3.4*	2.4	5.3*	4.0*	5.3*	4.6*	2.7	3.8*	3.6
PR_1_ –Curitiba, Paraná	1													
PR_2_ –Maringá, Paraná	1													
PR_3_—Santa Terezinha do Itaipu, Paraná	4													
PR_4_—Pato Branco, Paraná	1													
PR_5_—Foz do Iguaçu, Paraná	70	0.02	0.18	1.0	1.0	1.0	1.7	1.9*	2.8	2.7	1.0	1.0	1.9	1.6
PY–Paraguay	10	0.08	-0.02	1.0	1.0	1.0	2.0	1.0	2.0*	3.0	1.0	1.0	3.0	1.6
SC_1_—São Miguel do Oeste, Santa Catarina	7	0.31	1.00*	1.7	1.7	1.3	1.0	1.6*	1.0	1.0	1.0	1.0	1.0	1.2
SC_2_ –Descanso, Santa Catarina	9	0.00	1.00	1.0	1.5	1.0	1.0	1.4	#	1.0	#	1.0	1.0	1.1
SE–Aracaju, Sergipe	1													
SP_1_ –Bauru, São Paulo	3													
SP_2_ –Andradina, São Paulo	1													
TO–Palmas, Tocantins	2													
Total	132	0.07	0.46	1.1*	1.2*	1.7*	1.2	1.3*	1.6*	1.6*	1.3*	1.1*	1.4*	1.3

* Represents deviance from Hardy-Weinberg Equilibrium and significant Fis values. Gene diversity and Fis values are only presented for populations with more than 5 inividuals.

** Represents monomorphic populations.

# represents populations with missing data for a locus.

The isolates from other regions of Brazil were acquired from the Molecular Biology Laboratory of the Graduate Program in Bioprocess Engineering and Biotechnology of Universidade Federal do Paraná (UFPR). Additionally, 10 samples from Asunción (Paraguay, PY) were provided by the Laboratorio de Medicina Tropical of Instituto de Investigaciones en Ciencias de la Salud of Universidad Nacional de Asuncion, and seven samples from the Old World (MON-1 from France, Spain and Portugal, MON-24 from Algeria, MON-98 from Egypt, MON-108 from France and MON-198 from Spain) were kindly provided by the Molecular Ecologie Laboratory of the Medecine Faculty of the University of Montpelier, France.

### Ethics statement

The collection of human sampling was conducted in accordance with the International Ethical Guidelines for Biomedical Research in Humans. The samples were taken by the doctors. In addition, ethical approval was obtained from the Universidade Federal do Paraná Ethical Committee (number 684.244) and we complied with the minimum requirements of the Southern Common Market Treaty (Mercosur), Resolution No. 129/96. All individuals have signed the free consent clause indicating that they agree to use this sample. For dogs, all procedures were carried out in strict compliance with the rules defined by the National Council for the Control of Animal Experiments (CONCEA). Every effort was made to minimize suffering of the dogs. The work was approved by the Ethics Committee of the Federal University of Paraná (protocol number 044/2014). The owners have signed a consent form for the use of the samples.

### Microsatellite genotyping and population genetic analysis

Fourteen microsatellite markers (Li46-67, Li41-56, Li71-7, Li71-33, Li23-41, Li22-35, Lm2TG, Lm4TA, Li45-24, CS20, Li71-5/2, TubCA, List7031, List7039) described by Jamjoom et al. [58], Ochsenreither et al. [59] and Kuhls et al. [60] were selected to assess the genetic profile of the populations in central-southern Brazil.

The 10 μL PCR reactions were performed with 10x buffer, 1.5 mM MgCl_2_, 0.2 mM dNTP, 0.3 units of Platinum Taq DNA Polymerase (Invitrogen), 0.3 pmol of fluorescence conjugated forward primer (0.5 pmol for Li23-41, Li22-35, Lm4TA, Li45-24 and List7039) and the same quantity of the reverse primer, 10 ng of DNA template (5 ng for the loci Li71-7 and Lm2TG) and ultrapure water to complete the final volume. The PCR cycles were set to run for 3 min at 95°C for initial denaturation; 35 cycles of 30 s at 95°C for denaturation; 60 s at 50°C (Li46-67, Li41-56, Li71-7 and Li71-33), 52°C (Li23-41 and Li22-35), 54°C (Lm4TA and Li45-24), 55°C (Lm2TG), 56°C (CS20, Li71-5/2 and List7039) and 58°C (TubCA and List7031) for primer annealing, and 60 s at 72°C for DNA extension; and a final extension at 72°C for 60 min. The amplified products were genotyped in an automated capillary sequencer ABi 3130 (Applied Biosystems). The amplification of the 14 microsatellite markers and assessment of their fragment size were performed using Gene Marker V2.4.2 (SoftGenetics).

The presence of null alleles, allele dropout and scoring errors was analyzed with Micro-Checker 2.2.3 [61]. The presence of loci under selection was assessed in the BayeScan v2.1 [62] only for populations with more than five individuals. Hardy-Weinberg disequilibrium, diversity (gene diversity, Ho and He) and genetic differentiation (FST and AMOVA, only for populations with more than five strains) analyses were performed using the software Arlequin [63]. Allelic richness was calculated in FSTAT 2.9.3.2 [64]. The critical p value was corrected using the B-Y method [65] in analyses with multiple comparisons. The probable number of genetic populations was assessed using the assign method implemented in STRUCTURE 2.3.3 [66] with three runs for each *K* (K between 1 and 8), composed of a burn-in period of 500,000 itinerations and 5,000,000 Markov Chain Monte Carlo (MCMC) iterations, and no-admixture model. The ad hoc method of Evanno et al. [67], implemented on the online tool Structure Harvester [68], was used to assess the most likely value of K. However, the main assumptions of Structure Analysis are that the population present Hardy-Weinberg and linkage equilibrium, while species of *Leishmania* species frequently deviate from these assumptions (see the Results section and [21, 60, 69]) regarding caution in interpreting Structure Analysis results for *Leishmania* spp.). Thus, we also assigned strains of *L*. *(L*.*) infantum* using Discriminant Analysis of Principal Components (DAPC), which is free from the assumptions of H-W and linkage equilibrium [70]. This analysis was performed using the package 'adegenet' [71] in R 3.5.0 software (R development core team [72]). The optimum number of retained PCs was assessed using both α-score and cross-validation, while the numbers of clusters was chosen based on the results of structure analysis. Due its more flexible assumptions, the results of the DAPC were preferably used to assess the dispersion of *L*. *(L*.*) infantum* in central-southern Brazil. Strains with posterior probability of belong to a cluster higher than 0.80 in the DACP analysis were assigned to that cluster, while strains with posterior probability of belong to a cluster lower than 0.80 remained undetermined. Subestructuration within the clusters was assessed in 3 runs of 5,000,000 MCMC (burn-in of 500,000 iterations) with K between 1 and 4 in STRUCTURE for each cluster.

### First records of VL in cities of central-southern Brazil

The dispersion of *L*. *(L*.*) infantum* in central-southern Brazil was assessed using historical data of the first records of VL cases in dogs and humans in each city of the states of the region (Mato Grosso (MT), Mato Grosso do Sul (MS), São Paulo (SP), Goiás (GO), Minas Gerais (MG), Rio de Janeiro (RJ), Espírito Santo (ES), Paraná (PR), Santa Catarina (SC) and Rio Grande do Sul (RS). Additionally, cases reported in neighboring countries (Bolivia (BO), Argentina (AR), Paraguay (PY), Uruguay (UR)) were also added to the database to assess possible dispersion of the parasite from these countries. For this, a search was performed for publications available in the Scopus, PubMed, Google Scholar, and Scielo portals between 1913 and 2017 using the following keywords: “first case visceral leishmaniasis”, “visceral leishmaniasis in dogs”, “visceral leishmaniasis in human” or “*Leishmania infantum*". Descriptions of errant dogs were not considered due to uncertainty regarding origin (autochthonous or allochthonous). Records of autochtonous human VL cases in the SINAN database (Sistema de Informação de Agravos de Notificação, available in http://portalsinan.saude.gov.br/) were also considered. The SINAN is the database that presents the records of diseases with mandatory notification in Brazil, includinhg hVL. The hVL cases recorded in the SINAN ranges between 2001 and 2017. All cases were categorized into five ranges of years according the following events: 1. 1913 to 1980: population migration from the Northeast Region to central-southern Brazil (see [73–75]); 2. 1981 to 1997: beginning of rural exodus, with migration of people and their animals from rural to urban areas (see [74–76]); 3. 1998 to 2005: construction of the Bolivia-Brazil gas pipeline and migration of employees and their pets to the states of Mato Grosso do Sul (MS) and São Paulo (SP); epidemics of VL to large cities in the states of São Paulo (SP), Minas Gerais (MG) and Mato Grosso do Sul (MS) (e.g. [4, 35, 36, 49, 77–79]); 4. 2006 to 2010: first VL cases registered Argentina and in the state of Rio Grande do Sul, and dispersion from cities in Paraguay (e.g. [9,38,80–82]); 5. 2011 to 2018: Dispersion of VL cases in South Brazil (e.g. [39, 40, 42–45, 83]).

## Results

### Population genetic analysis

Among the 14 microsatellite markers assessed, the loci List 7031, Li 41–56, Li 45–24 and TubCA exhibited recurrent evidence of null alleles for some populations and were thus removed from further analyses. No loci presented evidence of balancing or positive selection. Greater allelic diversity was observed in Brazilian populations from Campo Grande (MS_2_) and Foz do Iguaçu (PR_5_), while the populations from Foz do Iguaçu and Paraguay (PY) had greater intra-population allelic richness (see Table 1).

The AMOVA (performed only with populations with more than five strains genotyped), revealed that 62% of the genetic variation of populations is at the inter-population level (FST = 0.617, p value = 0.000). Pairwise genetic differentiation found significant genetic differentiation (p < 0.017 after B-Y correction between Asunción (PY) and São Miguel do Oeste (SC_1_), Asunción (PY) and Descanso (SC_2_), São Miguel do Oeste (SC_1_) and Belo Horizonte (MG), Descanso (SC_2_) and Belo Horizonte (MG), São Miguel do Oeste (SC_1_) and Foz do Iguaçu (PR_5_), and Descanso (SC_2_) and Foz do Iguaçu (PR_5_) (Table 2).

**Table 2:** Pairwise genetic differentiation (FST) and their significance (in parenthesis) between *Leishmania infantum* populations from central-southern Brazil and Paraguay (PY). Only populations with more than five strains were used.

Population	PY–Asunción	MG– Belo Horizonte	PR_5_ – Foz do Iguaçu	SC_1_ – São Miguel do Oeste
MG—Belo Horizonte	0.000 (0.99)			
PR_5_—Foz do Iguaçu	0.027 (0.21)	0.000 (0.99)		
SC_1_—São Miguel do Oeste	0.677 (0.00)*	0.780 (0.00)*	0.877 (0.00)*	
SC_2_ –Descanso	0.822 (0.00)*	0.950 (0.00)*	0.921 (0.00)*	0.017 (0.67)

* represents significant values after B-Y correction (p < 0.017).

**Fig 1:**
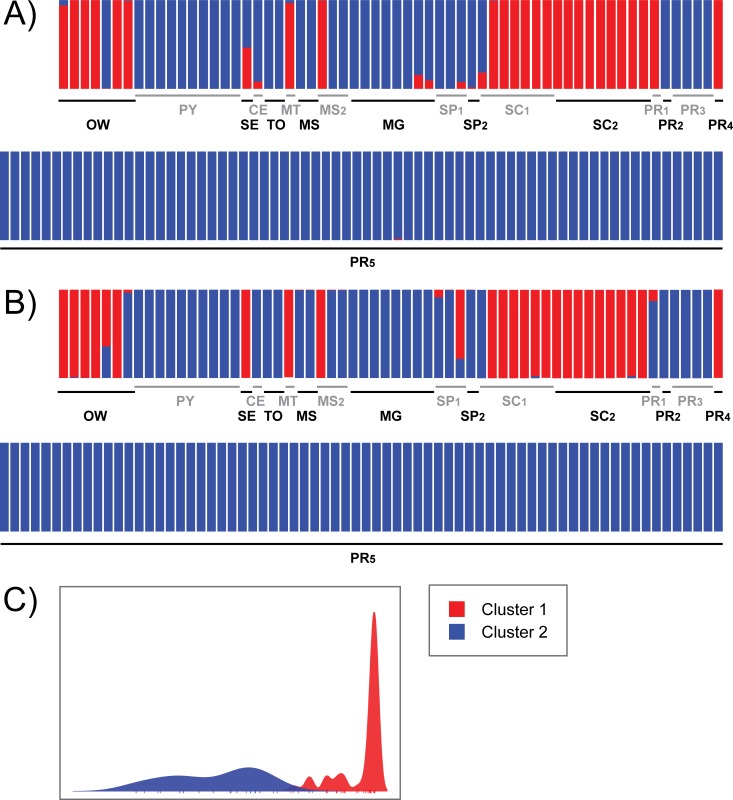
**Genetic assignment of 132 strains of *Leishmania infantum* from 18 populations of central-southern Brazil, Paraguay and the Old World, using Structure (A) and DAPC (B) with K = 2, and the discriminant function of the DAPC analysis (C).** Colors represent the genetic assigment of the strains of each population. Red: Cluster 1; green: Cluster 2.1; blue: Cluster 2.2. OW–Old World (strains belonging to MON-1 from France, MON-108 from France, MON-1 from Spain, MON-198 from Spain, MON-1 from Portugal, MON-24 from Algeria, MON-98 from Egypt); PY–Asunción, Paraguay; Brazilian populations (presented in the format Code–City, State): SE–Aracaju, Sergipe; CE–Fortaleza, Ceará; TO–Palmas, Tocantins; MT–Rondonópolis, Mato Grosso; MS_1_ –Três Lagoas, Mato Grosso do Sul; MS_2_ –Campo Grande, Mato Grosso do Sul; MG–Belo Horizonte, Minas Gerais; SP_1_ –Bauru, São Paulo; SP_2_ –Andradina, São Paulo; PR_1_ –Curitiba, Paraná; PR_2_ –Maringá, Paraná; PR_3_ –Santa Terezinha do Itaipu, Paraná; PR_4_ –Pato Branco, Paraná; PR_5_ –Foz do Iguaçu, Paraná; SC_1_ –São Miguel do Oeste, Santa Catarina; SC_2_ –Descanso, Santa Catarina. Gray and black names are only for make reading easier.

The ad hoc method of Evanno et al. (2005) [67] supported two (ΔK: 43.4) as the most probable number of clusters of the assignment analysis implemented in Structure, followed by three (ΔK: 27.0). Since both analyses were informative, the results of K = 2 and K = 3 are presented. The Structure and DACP analyses with 2 clusters indicated that the populations of São Miguel do Oeste (SC_1_, except one strain), Descanso (SC_2_), Aracaju (SE, undetermined in Structure analysis), Rondonópolis (MT), Curitiba (PR_1,_ only in Structure analysis), Pato Branco (PR_4_), and one strain from Campo Grande (MS_2_) compose a cluster (named Cluster 1), while the other populations from South America compose the other cluster (named Cluster 2) (Fig 1).

**Fig 2:**
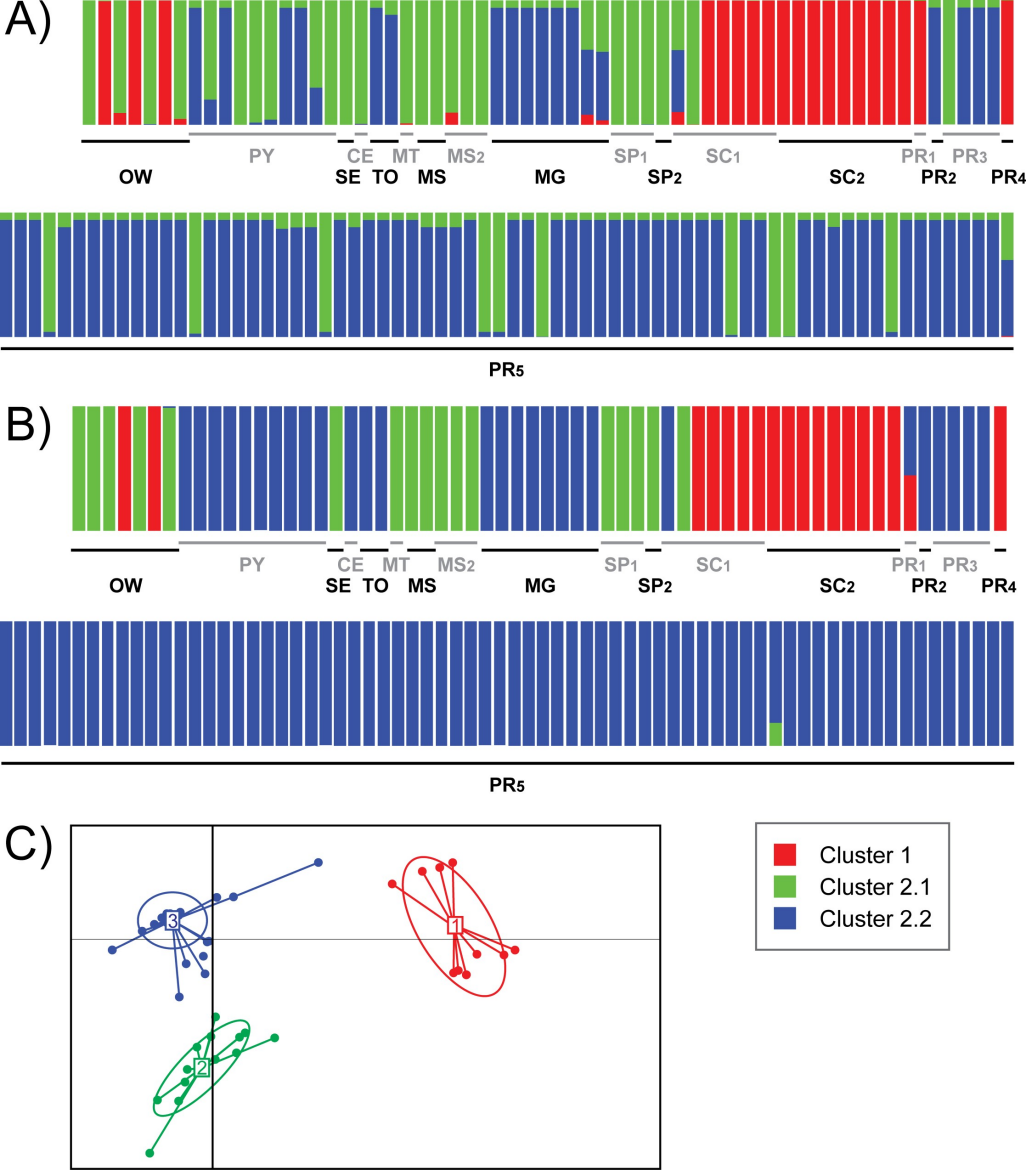
**Genetic assignment of 132 strains of *Leishmania infantum* from 18 populations of central-southern Brazil, Paraguay and the Old World using Structure (A) and DAPC (B) with K = 3, and the discriminant function of the DAPC analysis (C).** Colors represent the genetic assigment of the strains of each population. Red: Cluster 1; green: Cluster 2.1; blue: Cluster 2.2. OW–Old World (strains belonging to MON-1 from France, MON-108 from France, MON-1 from Spain, MON-198 from Spain, MON-1 from Portugal, MON-24 from Algeria, MON-98 from Egypt); PY–Asunción, Paraguay; Brazilian populations (presented in the format Code–City, State): SE–Aracaju, Sergipe; CE–Fortaleza, Ceará; TO–Palmas, Tocantins; MT–Rondonópolis, Mato Grosso; MS_1_ –Três Lagoas, Mato Grosso do Sul; MS_2_ –Campo Grande, Mato Grosso do Sul; MG–Belo Horizonte, Minas Gerais; SP_1_ –Bauru, São Paulo; SP_2_ –Andradina, São Paulo; PR_1_ –Curitiba, Paraná; PR_2_ –Maringá, Paraná; PR_3_ –Santa Terezinha do Itaipu, Paraná; PR_4_ –Pato Branco, Paraná; PR_5_ –Foz do Iguaçu, Paraná; SC_1_ –São Miguel do Oeste, Santa Catarina; SC_2_ –Descanso, Santa Catarina. Gray and black names are only for make reading easier.

The Structure and DACP analysis with K = 3 divided Cluster 2 into two other clusters (named Clusters 2.1 and 2.2) (Fig 2). The Cluster 1 contained only populations São Miguel do Oeste (SC_1_, except two strains), Descanso (SC_2_), Curitiba (PR_1_, undetermined in the DACP analysis; and Pato Branco (PR_4_), as well as MON-24 from Algeria, MON-108 from France and MON-198 from Spain. Cluster 2.1 encompassed the strains from Aracaju (SE), Rondonópolis (MT), Campo Grande (MS_1_), Três Lagoas (MS_2_), Bauru (SP_1_), Andradina (SP_2_), a strain from Descanso (SC_1_), MON-1 from France, Spain and Portugal, and MON-98 from Egypt. The Structure analysis also assigned five strains from Asunción (PY), one from Fortaleza (CE), one from Santa Terezinha de Itaipu (PR_3_), and ten from Foz do Iguaçu (PR_5_) to Cluster 2.1. The strains from populations Asunción (PY), Fortaleza (CE), Palmas (TO), Belo Horizonte (MG), Maringá (PR_2_), Santa Terezinha de Itaipu (PR_3_), Foz do Iguaçu (PR_5_) and a strain from São Miguel do Oeste (SC_1_) were assigned to Cluster 2.2. In the subestructuration analysis, each cluster presented a cohesive genetic group, with no signal of genetic subestructuration.

### First records of VL in cities of central-southern Brazil

**Fig 3:**
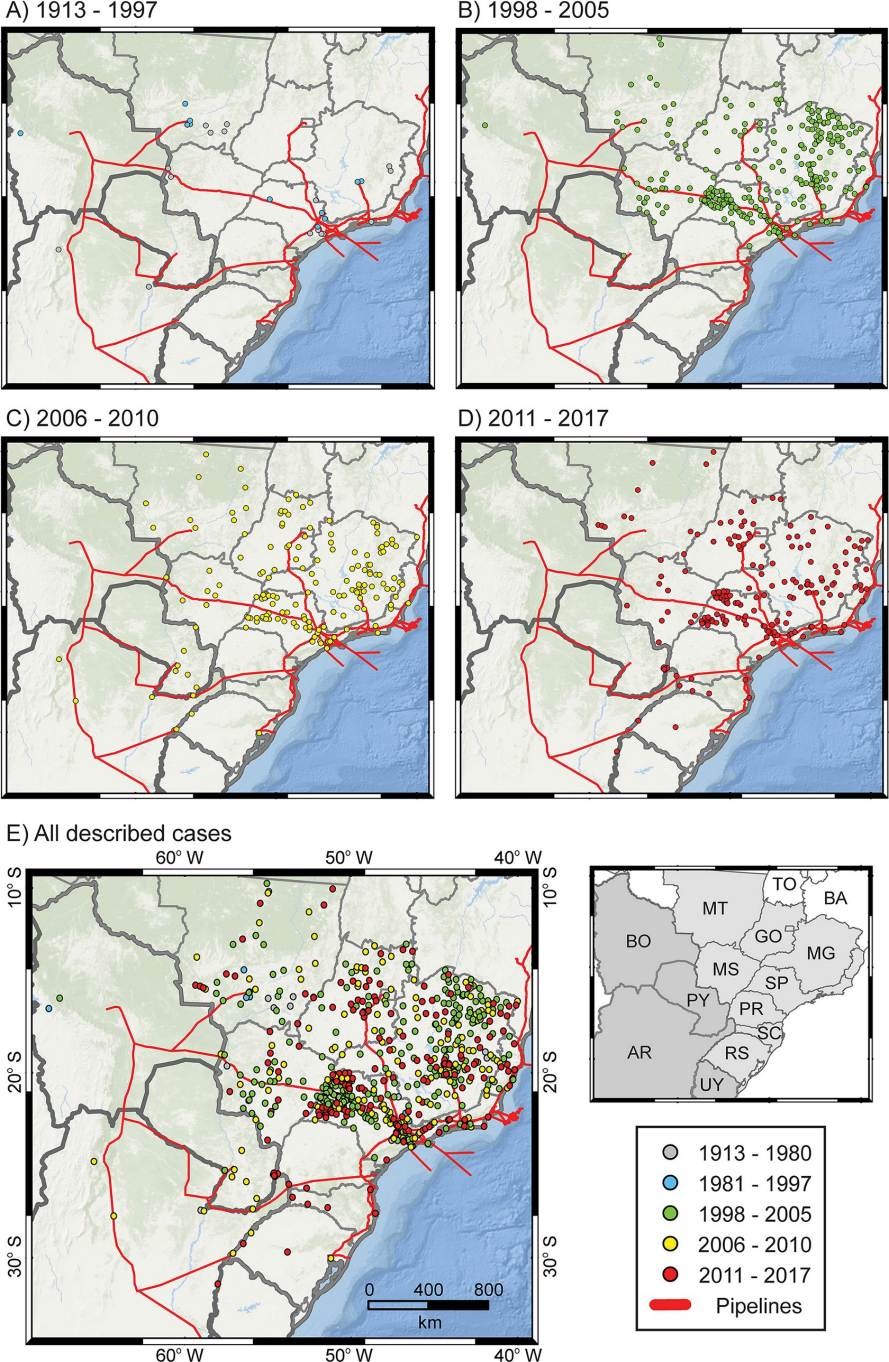
**Spatial distribution of the first VL reports in cities of central-southern Brazil between 1913 and 1980 (A—pink), 1981 and 1997 (A—blue), 1998 and 2005 (B), 2006 and 2010 (C), 2011 and 2017 (D), and all cases described (E).** Countries in dark gray are those assessed in this study: AR: Argentina; BO: Bolivia; PY: Paraguay; UY: Uruguay. Brazilian states in light gray are those assessed in this study: GO: Goiás; MG: Minas Gerais; MS: Mato Grosso do Sul; MT: Mato Grosso; PR: Paraná; RS: Rio Grande do Sul; SC: Santa Catarina; SP: São Paulo. The Brazilian states Bahia (BA) and Tocantins (TO) (in white) were not assessed in the first VL reports research. The paths of the pipelines are based on EPE [84]. The map in Fig 3 was constructed in the ArcMap 10.2.2, using the World Ocean Base (from ESRI, DeLorme, The General Bathymetric Chart of the Oceans—GEBCO, National Oceanic and Atmospheric Administration–NOAA, National Geophysical Data Center—NGDC, and other contributors) as basemap.

The research for first records of VL cases in dogs and humans resulted in 52,029 articles published between 1913 and 2018, of which 350 were pre-selected due their epidemiological information or the report of the first case of VL in cities of central-southern South America. Among these articles, 55 were selected due to human or canine VL reports in Bolivia, Paraguay, Argentina, Uruguay or Brazil. Cases of VL were described for 672 cities (Table 3 and Fig 3), of which 611 were the first record in humans (hVL) and 61 the first record in dogs (cVL). The SINAN database contained 456 human VL records between 2001 and 2017, while the literature revealed 216 cVL and hVL cases between 1913 and 2017.

**Table 3:** Number of cities per country (in bold), and per central-southern Brazilian state (in italic), with record of the first case of Visceral Leishmaniasis in each period accessed by SINAN, literature (Lit) and Total. Blank values represent non available values. SINAN database presented VL notifications only for Brazilian cities, between 2001 and 2017.

Country– States	1913–1980			1981–1997			1998–2005			2006–2010			2011–2018			Total		
	SINAN	Lit	Total	SINAN	Lit	Total	SINAN	Lit	Total	SINAN	Lit	Total	SINAN	Lit	Total	SINAN	Lit	Total
**Bolivia**		**0**	**0**		**1**	**1**		**1**	**1**		**1**	**1**		**0**	**0**		**3**	**3**
**Argentina**		**2**	**2**		**0**	**0**		**0**	**0**		**3**	**3**		**1**	**1**		**6**	**6**
**Paraguay**		**0**	**0**		**0**	**0**		**1**	**1**		**5**	**5**		**3**	**3**		**9**	**9**
**Uruguay**		**0**	**0**		**0**	**0**		**0**	**0**		**0**	**0**		**1**	**1**		**1**	**1**
**Brazil**		**14**	**14**		**10**	**10**	**225**	**56**	**281**	**106**	**58**	**164**	**125**	**59**	**184**	**456**	**197**	**653**
*Espirito Santo (MS)*		0	0		0	0	9	0	9	2	0	2	4	0	4	15	0	15
*Goiás (GO)*		0	0		0	0	17	0	17	15	10	25	22	0	22	54	10	64
*Mato Grosso (MT)*		4	4		4	4	17	1	18	10	0	10	11	1	12	38	10	48
*Mato Grosso do Sul (MS)*		2	2		0	0	25	2	27	9	0	9	7	4	11	41	8	49
*Minas Gerais (MG)*		2	2		2	2	103	3	106	46	11	57	47	2	49	196	20	216
*Paraná (PR)*		0	0		0	0	0	0	0	0	0	0	1	4	5	1	4	5
*Rio de Janeiro (RJ)*		1	1		0	0	3	1	4	3	1	4	8	1	9	14	4	18
*Rio Grande do Sul (RS)*		0	0		0	0	0	0	0	0	3	3	2	0	2	2	3	5
*Santa Catarina (SC)*		0	0		0	0	0	0	0	0	0	0	1	4	5	1	4	5
*São Paulo (SP)*		5	5		4	4	51	49	100	21	33	54	22	43	65	94	134	228
**Total**	**0**	**16**	**16**	**0**	**11**	**11**	**225**	**58**	**283**	**106**	**67**	**173**	**125**	**64**	**189**	**456**	**216**	**672**

Between 1913 and 1980 (Fig 3A), VL was described for the first time in 16 cities (15 hVL, 1 cVL) in Argentina (Northwest and Chaco regions) and Brazil (Central-West and Southeast regions). The number of cases remained low between 1981 and 1997 (Fig 3A), with first records of VL in 11 cities (8 hVL, 3 cVL), including 10 new records in Brazil and one in Bolivia.

Between 1998 and 2005, the first years with records in SINAN database, the number of cities with their first records of VL increased between 1998 and 2005 (Fig 3B), with 283 (270 hVL, 13cVL) from Paraguay (1), Bolivia (1) and Brazil (281), with the Brazilian states of Minas Gerais (106) and São Paulo (100) containing most these records. The new records of VL in the state of São Paulo were registered in cities close to the Bolivia-Brazil gas pipeline, most of them described in the literature, while for Minas Gerais they were registered mainly in the SINAN database in cities close to the states in the Northeast Region of the country (i.e. Bahia) and near the state capital Belo Horizonte.

Between 2006 and 2010 (Fig 3C), 173 cities in Argentina (3), Paraguay (5), Bolivia (1) and Brazil (164) had their first VL cases (141 hVL, 32 cVL). In Brazil, VL spread to cities northward and southward from the cities in the São Paulo state that is close to the Bolivia-Brazil gas pipeline and had their first records in previous period. Moreover, the first records of VL for the South Region of Brazil were during this period in three cities in the state of Rio Grande do Sul. New records of VL in Argentina occurred in cities close to gas pipelines and the frontiers with Paraguay and Brazil (i.e. Rio Grande do Sul state).

Between 2011 and 2017 (Fig 3D), 189 new cities recorded VL (177 hVL, 12 cVL), three in Paraguay, one in Argentina, one in Uruguay and 184 in Brazil. The number of new cities with cases increased in the South Region of Brazil, with five in Paraná, five in Santa Catarina and two in Rio Grande do Sul). In the state of São Paulo, VL continued its spread to cities northward and southward of the Bolivia-Brazil gas pipeline.

## Discussion

**Fig 4:**
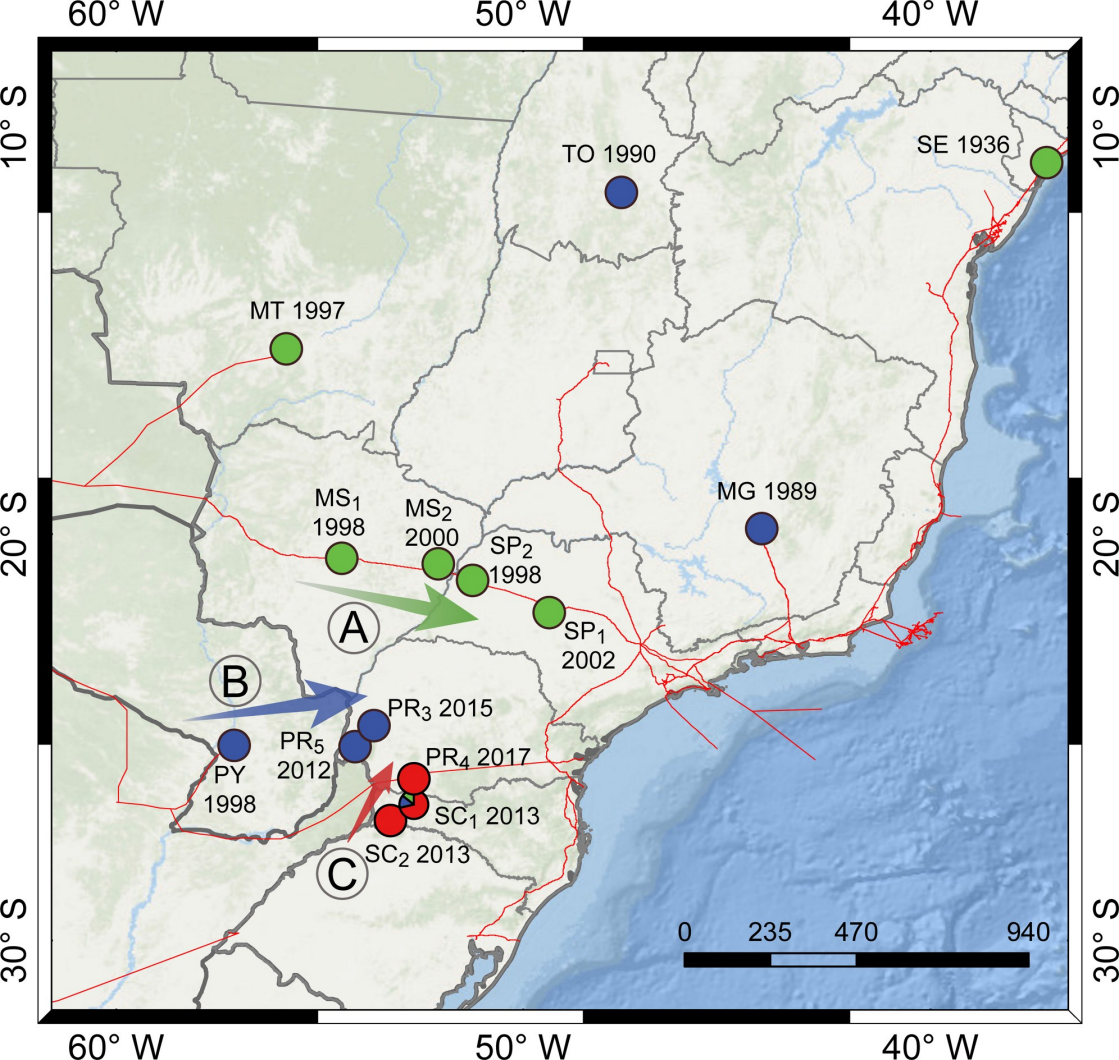
**Dispersion routes (arrows) of *L*. *(L*.*) infantum* from central-southern supported by historical reports of Visceral Leishmaniasis and by the genotyping 14 populations Brazil and Paraguay: A. Dispersion west-to-east along the Bolivia-Brazil gas pipeline; B. Dispersion from Paraguay (PY) to border cities of the state of Paraná (PR); C. Dispersion of a new cluster from Santa Catarina (SC) to Paraná (PR).** Circle color represents the genetic assignment of the strains of each population as assessed by DAPC analysis (i.e. red: Cluster 1; green: Cluster 2.1; blue: Cluster 2.2), while arrow color represents the hypothesized cluster that dispersed along that route. PY (Asunción, Paraguay) and Brazilian populations (presented in the format Code–City, State): (SE (Aracaju city, Sergipe state); TO (Palmas, Tocantins); MT (Rondonópolis, Mato Grosso); MS_1_ (Três Lagoas, Mato Grosso do Sul); MS_2_ (Campo Grande, Mato Grosso do Sul); MG (Belo Horizonte, Minas Gerais); SP_1_ (Bauru, São Paulo); SP_2_ (Andradina, São Paulo); PR_3_ (Santa Terezinha do Itaipu, Paraná); PR_4_ (Pato Branco, Paraná); PR_5_ (Foz do Iguaçu, Paraná); SC_1_ (São Miguel do Oeste, Santa Catarina); SC_2_ (Descanso, Santa Catarina). Strains from Curitiba (PR_1_) and Maringá (PR_4_) are not presented since they are allochthonous cVL cases. Red lines represent pipelines, based on EPE [84]. The map in Fig 4 was constructed in the ArcMap 10.2.2, using the World Ocean Base (ESRI, DeLorme, The General Bathymetric Chart of the Oceans—GEBCO, National Oceanic and Atmospheric Administration–NOAA, National Geophysical Data Center—NGDC, and other contributors) as basemap.

The results of the microsatellite and historical report analyses support three dispersion routes for *L*. *(L*.*) infantum* in central-southern Brazil, as follow: A) Dispersion from west to east along the Bolivia-Brazil gas pipeline between 1998 and 2005 (A in the Fig 4); B) Dispersion from Paraguay to Brazilian cities at the triple border after 2012 (i.e. Foz do Iguaçu and Santa Terezinha de Itaipu) (B in the Fig 4); C) The emergence of a new cluster in western Santa Catarina State (i.e. the cities of São Miguel do Oeste and Descanso) in 2013, and its dispersion to southern Paraná State (Pato Branco) (C in the Fig 4). The evidence supporting each reconstructed route is provided below.

The first putative dispersion route of VL into central-southern Brazil probably followed the construction of the Bolivia-Brazil gas pipeline between 1998 and 2005 (Fig 4B), first in cities close to the gas pipeline, and then to the north and to the south of these cities. This dispersion route has previously been suggested by studies based on historical cases in Mato Grosso do Sul [49] and São Paulo [55], on historical and molecular data [79] and on microsatellite markers [51]. Our microsatellite data also supported this hypothesis. Our Cluster 2.1, which probably represents cluster POP-3 of Ferreira et al. [51], is present in populations from Mato Grosso do Sul (Campo Grande–MS_1_ and Três Lagoas–MS_2_) and São Paulo (Bauru–SP_1_ and Andradina–SP_2_). The construction of the Bolivia-Brazil gas pipeline started in 1998 in Bolivia and expanded into Mato Grosso do Sul (from Corumbá to Três Lagoas), São Paulo (from northwest, close to Andradina, to the southeast), Rio de Janeiro and Minas Gerais. During this construction, the displacement of employees and their pets from Corumbá (an endemic area) to other cities of Mato Grosso do Sul and neighboring states along the course of gas pipeline may have dispersed the parasite into the Southeast Region [49]. Moreover, the deforestation and ecological imbalance resulting from the construction itself may have favored the dispersion of the vector *Lu*. *longipalpis* into urban areas [85]. Alternatively, Oliveira et al. [36] found no correlation between the date of vector detection and confirmation of the autochthonous cVL and hVL, with the construction of the Bolivia–Brazil gas pipeline, and they found positive correlation between the presence of the Marechal Rondon highway and of transverse highways with the presence of the vector in the São Paulo State. Highways permit the transportation of people, host and vectors, including those infected with infectious diseases. Once the Bolivia-Brazil gas pipeline presets high collinearity with the BR-272 (in Mato Grosso do Sul) and the Marechal Rondon highway (in the São Paulo State), isolating the influence of these highways from those of the Bolivia-Brazil gas pipeline is difficult. In this way, the influence of the highways, or even a composed influence of highways and gas pipelines, should not be discarded.

Our results also support a possible dispersion of *L*. *(L*.*) infantum* from Bolivia to Rondonópolis (MT) though a secondary gas pipeline from Bolivia to Cuiabá (MT) (182 km from Rondonópolis), since the same cluster (Cluster 2.1) was found in this city and other cities of Mato Grosso do Sul and São Paulo. This result opposes that of Ferreira et al. [51], who found 86% of the 11 *L*. *(L*.*) infantum* strains from Cuiabá (MT) and 3 strains from Rondonópolis (MT) to belong to the POP-2 cluster (not found in our study), and 14% of belonging to the POP-1 cluster (our cluster 2.2). Thus, our samples are the first to record the POP 3 (cluster 2.1) in Mato Grosso state.

The seccond dispersion hypothesis represents the dispersion of *L*. *(L*.*) infantum* from Paraguay to Brazilian cities close to the triple border between Argentina-Brazil-Paraguay (Fig 4C). VL was first described in the Asunción Paraguay in 1998, and then was found to have dispersed to other cities in the country between 2006 and 2010, likely reaching the triple border around 2012. Supporting this, the Cluster 2.2 is the most prevalent cluster in Paraguay, Foz do Iguaçu and Santa Terezinha de Itaipu. On the Brazilian side of the triple border, Foz do Iguaçu borders Argentina and Paraguay and it is considered a potential place for VL dispersion due the intense flow of people and environmental conditions suitable for the emergence of *Lu*. *longipalpis* and VL (see Thomaz-Soccol et al. [3, 45] for details). The first description of the vector (*Lu*. *longipalpis*) in this region was in 2012, the first cVL in 2013 and the first hVL in 2016, with increasing number of cases in subsequent years [41–44]. Currently, Thomaz-Soccol et al. [45] reported that about 24% of the dogs in Foz do Iguaçu possess cVL, and that the parasite and the vector are widely present in Foz do Iguaçu and the neighboring city Santa Terezinha de Iguaçu. Thus, Foz do Iguaçu is now considered an endemic city for *L*. *(L*.*) infantum* due to the generalized distribution of seropositive dogs, abundance of *Lu*. *longipalpis* and human cases in all areas of the city.

Moreover, Ferreira et al. [51] described that the Paraguayan populations of *L*. *(L*.*) infantum* (POP-1, probably our Cluster 2.2) are also present in populations from Rio Grande do Sul state (Uruguaiana, São Borja and Santa Maria). These results suggest that this cluster may have been widely distributed in southern South America (i.e. Paraguay, South Brazil), including Argentina and Uruguay. Thus, an alternative route for the dispersion of *L*. *(L*.*) infantum* to Foz do Iguaçu may have been the spread from Argentina to the Triple Border, to cities of the state of Rio Grande do Sul and to Uruguay. Similar to Paraguayan cities, Argentinian cities of the Mesopotamia region had VL cases since 2006, thus earlier than Foz do Iguaçu. Genotyping of strains from Argentina would certainly help to test this hypothesis.

Finally, the third dispersion of *L*. *(L*.*) infantum* in the South Region of Brazil is represented by a new cluster in western Santa Catarina State. The first autochthonous cases of cVL in this state were in Florianópolis in 2010 and in the western region in 2013 (São Miguel do Oeste and Descanso) [82]. These *L*. *(L*.*) infantum* strains were assigned to a new cluster (here named cluster 1), and probably dispersed to southern Paraná State (i.e. Pato Branco) (Fig 4D). The allochthonous cVL case described for Curitiba in 2004 (a dog captured off the street, see Thomaz-Soccol et al. [85] for more details) was also assigned in this cluster. The origin of this cluster is still unclear, but it is genetically similar to MON-24 from Algeria, MON-108 from France and MON-198 from Spain. Therefore, this cluster may represent a new contemporary introduction of *L*. *(L*.*) infantum* from the Old World.

Molecular markers, including microsatellite markers, have been used for a wide range of groups to assess genetic structure, dispersion and to assign individuals to populations [86,87]. However, the usefulness of these markers is limited when the diversity is low, which is a common feature of invasive species, as is the case for *L*. *(L*.*) infantum* in the New World [18,19,29,48,49,58,59]. Moreover, most sample sizes of the present study, as well as that of other investigations (e.g. [21,48]), are low and may not represent the entire diversity of each population, and thus limit statistical power for testing more specific hypothesis about the genetic structure and dispersion of *L*. *(L*.*) infantum* in the Brazil. Collaboration among researchers with the exchange of samples is essential for increasing sample sizes and the distribution of sampling to better understand the dynamics of this and other parasites in Brazil. This information is vital for developing public policies for effective control and restraint of VL dispersion.

Integrating molecular markers with other forms of data, like historical reports, is important and could increase the reliability of reconstructions and hypotheses of dispersal of *L*. *(L*.*) infantum*. However, some inferences using these data must be done with care since limitations on the notification of the cases may bias analyses. First, most of the VL cases found in our research were reported in SINAN, and this database has not presented the hVL cases before 2001. Thus, some cities that SINAN reported the presence of VL in and after 2001 probably had already presented cases of VL before 2001. This factor may explain the increase in the number of hVL cases between 1998 and 2005, especially in Minas Gerais. Second, hVL and cVL may often be reported in cities that are not the site of the original infection (allochthonous cases). Third, several cVL cases may not be diagnosed and/or described. Cities without health professionals trained in hVL diagnosis may have several non-recorded cases. These issues could explain the higher number of cases described in Minas Gerais and São Paulo than in neighbor states that should also have a great number of cities with VL cases (i.e. Mato Grosso, Mato Grosso do Sul, Goiás e Rio de Janeiro). These two states are among the top five states regarding health systems in Brazil (4^th^ and 5^th^, respectively), while their neighboring states rank much lower among the 27 Federal Units (26 states and the Federal District): Mato Grosso 11^th^, Goiás 13^th^, Mato Grosso do Sul 14^th^, and Rio de Janeiro 23^th^ [88]. Moreover, the few cases reported for neighboring countries is probably due to the absence of unified databases and/or an obligation to describe VL cases. Thus, approaches that integrate different data allow the body of information to be increased, thus reducing potential bias in analyses.

The dispersion routes proposed herein are the result of complex scenarios that include population migrations and environmental changes. The recent urban dissemination of VL in medium and large cities and the spread to other regions of Brazilian suggest worsening scenarios in the future. The movement of people and their infected dogs [89], and the lack of joint policies with countries bordering Brazil (approximately 8 thousand km of border) are risks for VL dispersion to South Region of Brazil. The dispersion routes identified herein should be considered in developing plans to efficiently control VL and avoid its further dispersion in central-south Brazil.

### Conclusion

In summary, our results highlight the need of the development of plans that efficiently avoid the dispersion of the visceral leishmaniasis in the central-southern Brazil that includes monitoring of this diseases and joint policies with countries bordering this Brazilian region.
